# HIV-1 Vpr Modulates Macrophage Metabolic Pathways: A SILAC-Based Quantitative Analysis

**DOI:** 10.1371/journal.pone.0068376

**Published:** 2013-07-12

**Authors:** Carlos A. Barrero, Prasun K. Datta, Satarupa Sen, Satish Deshmane, Shohreh Amini, Kamel Khalili, Salim Merali

**Affiliations:** 1 Department of Biochemistry, Temple University School of Medicine, Fels Institute, Philadelphia, Pennsylvania, United States of America; 2 Department of Neuroscience, Temple University School of Medicine, Philadelphia, Pennsylvania, United States of America; 3 Center for Neurovirology, Temple University School of Medicine, Philadelphia, Pennsylvania, United States of America; 4 Department of Biology, Temple University, Philadelphia, Pennsylvania, United States of America; Institute of Human Virology, Baltimore, United States of America

## Abstract

Human immunodeficiency virus type 1 encoded viral protein Vpr is essential for infection of macrophages by HIV-1. Furthermore, these macrophages are resistant to cell death and are viral reservoir. However, the impact of Vpr on the macrophage proteome is yet to be comprehended. The goal of the present study was to use a stable-isotope labeling by amino acids in cell culture (SILAC) coupled with mass spectrometry-based proteomics approach to characterize the Vpr response in macrophages. Cultured human monocytic cells, U937, were differentiated into macrophages and transduced with adenovirus construct harboring the Vpr gene. More than 600 proteins were quantified in SILAC coupled with LC-MS/MS approach, among which 136 were significantly altered upon Vpr overexpression in macrophages. Quantified proteins were selected and clustered by biological functions, pathway and network analysis using Ingenuity computational pathway analysis. The proteomic data illustrating increase in abundance of enzymes in the glycolytic pathway (pentose phosphate and pyruvate metabolism) was further validated by western blot analysis. In addition, the proteomic data demonstrate down regulation of some key mitochondrial enzymes such as glutamate dehydrogenase 2 (GLUD2), adenylate kinase 2 (AK2) and transketolase (TKT). Based on these observations we postulate that HIV-1 hijacks the macrophage glucose metabolism pathway via the Vpr-hypoxia inducible factor 1 alpha (HIF-1 alpha) axis to induce expression of hexokinase (HK), glucose-6-phosphate dehyrogenase (G6PD) and pyruvate kinase muscle type 2 (PKM2) that facilitates viral replication and biogenesis, and long-term survival of macrophages. Furthermore, dysregulation of mitochondrial glutamate metabolism in macrophages can contribute to neurodegeneration via neuroexcitotoxic mechanisms in the context of NeuroAIDS.

## Introduction

Human immunodeficiency virus type 1 (HIV-1) viral protein R (Vpr) is a small 96-amino acid multifunctional protein [1]–[6]. Vpr is essential for HIV-1 infection of macrophages since virus deficient in Vpr is less efficient in replication in macrophages [7]. Furthermore, extracellular Vpr can rescue replication of Vpr-deficient HIV strains in macrophages [8]. HIV-1 LTR activation by Vpr results in increased viral replication [9], [10]. Vpr-mediated transcriptional induction of HIV-1 involves interaction between Vpr with specific sequences that span the C/EBP and adjacent NFκB sites of HIV-1 LTR [11], and transcription factor, Sp1 [12], [13]. Vpr induces apoptosis in several cell types, including lymphocytes, monocytes, astrocytes, and neurons [14]–[19]. However, HIV-1 infected macrophages are resistant to apoptosis [20]. These observations suggest that Vpr modulates macrophage proteome to promote viral replication and induce anti-apoptotic pathways. This acquired anti-apoptotic phenotype may promote reservoir formation in this cell type. Therefore, analysis of the macrophage proteome in Vpr expressing macrophages can help to better understand mechanisms involved in HIV-1 replication and survival.

A variety of stable-isotope labeling strategies, such as isotope-coded affinity tag (ICAT), isobaric tags for relative and absolute quantitation (iTRAQ) and stable-isotope labeling by amino acids in cell culture (SILAC) coupled with mass spectrometry (MS)-based proteomics allows reliable identification and quantitative analysis of multiple proteins in complex samples [21]–[23]. We used SILAC, as a metabolic labeling method, since it is simple, efficient, and allows for almost complete heavy isotope incorporation in cells [23], [24]. To explore novel mechanisms underlying Vpr-mediated modulation of macrophage proteome, we employed LC-MS/MS, along with SILAC to assess quantitatively Vpr-induced perturbation of protein expression in U937 derived macrophages.

More than 600 proteins were quantified in SILAC coupled with LC-MS/MS measurement, among which 136 were significantly altered upon Vpr overexpression in macrophages. Importantly, we observed, for the first time, that Vpr-induced up-regulation of enzymes in the pyruvate metabolism, pentose phosphate pathway, and mitochondrial dysfunction.

## Materials and Methods

### Cell Line

The human monocytic cell line U937 was obtained from American Type Culture Collection (Manassas, VA).

### Chemicals and Antibodies

Heavy lysine and arginine ([^13^C_6_, ^15^N_2_]-L-lysine and [^13^C_6_]-L-arginine) were obtained from Cambridge Isotope (Andover, MA) and light amino acids (L-lysine and L-arginine) were obtained from Sigma-Aldrich (St. Louis, MO). All components of cell culture media were obtained from Life Technologies (CA) and protease inhibitor cocktail was obtained from Sigma-Aldrich (St, Louis, MO). SILAC DMEM Media was obtained from Pierce Biotechnology and the dialyzed FBS was purchased from HyClone (Logan, UT). Trypsin was purchased from Promega (Madison, WI). All the chemicals were HPLC-grade unless specifically mentioned. The antibodies against HK-1, HK-2, G6PD, PKM2 and Grb2 were obtained from Cell Signaling Technology (Danvers, MA); HIF-1 alpha antibody was obtained from BD Biosciences (San Jose, CA).

### Cell Culture

U937 cells were cultured in RPMI containing 10% FBS supplement with penicillin and streptomycin at 37°C in humidified atmosphere with 5% CO_2_.

### Construction of Recombinant Adenoviruses

To construct recombinant adenoviral vector harboring HIV-1 Vpr, Vpr cDNA from the dual-tropic (CCR5 and CXCR4) strain of HIV-1 89.6 [25] was used. Vpr cDNA (288 bp) was excised from pcDNA_3_-Vpr and cloned into the EcoRI and NheI sites of the adenovirus-shuttle plasmid pDC515 under the control of the murine cytomegalovirus promoter (purchased from Microbix Inc., Ontario, Canada). Adeno-Vpr recombinant shuttle containing Vpr sequence (pDC515-Vpr) was transfected into HEK-293 cells with pBHGfrt (del) E1, 3FLP, and a plasmid that provides adenovirus type 5 genome deleted in *E1* and *E3* genes. Plaques of recombinant adenovirus arising as a result of frt/FLP recombination were isolated, grown, and purified by cesium chloride density equilibrium banding. Empty shuttle plasmid, pDC515, was used to construct control adenoviral vector (Adeno-null, a virus without a transgene).

### Differentiation of U937 Cells into Macrophages and Culture in SILAC Media

SILAC DMEM media was supplemented with 10% dialyzed fetal bovine serum, 1% streptomycin/penicillin. The medium was then divided and supplemented with ^13^C_6_ L-arginine and ^13^C_6_, ^15^N_2_-L-lysine or normal L-arginine and L-lysine, to produce heavy or light SILAC medium, respectively. U937 cells were treated with 100 ng/ml of phorbal myristate acetate (PMA) for 3 hrs in complete RPMI medium at 37°C and then washed with 1XPBS and cultured for an additional 24 h in complete RPMI medium at 37°C. For SILAC experiments, the PMA differentiated cells were then grown in parallel in either light or heavy media for 5 days, with media replacement every 24 h.

### Transduction of SILAC Labeled Cells with Adenoviral Constructs

The PMA differentiated cells (macrophages) grown in SILAC media were then transduced with adenoviral stock corresponding to a multiplicity of infection (MOI) of five plaque-forming units per cell. The heavy labeled cells were transduced with Adeno-Vpr, while the light-labeled cells were transduced with Adeno-Null. The cells were harvested at 72 h post infection.

### Preparation of Protein Samples, 1-D SDS-PAGE Separation and In-gel Trypsin Digestion

Proteins were processed for gel electrophoresis-liquid chromatography-mass spectroscopy (GeLC-MS/MS) proteomics analysis. Total cell proteins were extracted from cells transduced with Adeno-Vpr and control cells using RIPA buffer (25 mM Tris•HCl pH 7.6, 150 mM NaCl, 1% NP-40, 1% sodium deoxycholate, 0.1% SDS). Protein quantification was performed using the method of Bradford (Bio-Rad Protein Assay). 60 µg of total proteins (30 µg “heavy” and 30 µg “light”) were diluted with Laemmli sample buffer (Bio-Rad) containing 5% β-mercaptoethanol. The mixture was heated for 5 min at 90°C and loaded onto 10% polyacrylamide gel. 1-D SDS-PAGE separation was performed using a mini Protean II system (BioRad) at 200 V for 45 min. Bands were visualized with Simply Blue Safe Stain and lanes were sliced into 11 sections, which were diced into ∼ 1×1 mm. After distaining with 50% v/v Acetonitrile (ACN) in 25 mM ammonium bicarbonate buffer (bicarbonate buffer), proteins within gel pieces were reduced with 10 mM DTT in bicarbonate buffer and alkylated by incubation with 50 mM iodoacetamide in bicarbonate buffer. After gel dehydration with 100% ACN, the gel pieces were covered with approximately 40 µL of 12.5 µg/mL trypsin in bicarbonate buffer. In gel digestion was done at 37°C for 12 h, trypsin was inactivated with formic acid at 2% final volume and peptides were extracted and clean-up using C18 Tip column (ZipTips®) as previously described [24].

### GeLC-MS/MS and Data Analysis

Peptides were dried in a vacuum centrifuge then resuspended in 30 µL of 0.1% v/v TFA/H2O. Peptide samples were loaded onto 2 µg capacity peptide traps (CapTrap; Michrom Bio-resources) and separated using a C18 capillary column (15 cm 75 mm, Agilent) with an Agilent 1100 LC pump delivering mobile phase at 300 nL/min. Gradient elution using mobile phases A (1% ACN/0.1% formic acid, balance H_2_O) and B (80% ACN/0.1% formic acid, balance H_2_O) was as follows (percentages for B, balance A): linear from 0 to 15% at 10 min, linear to 60% at 60 min, linear to 100% at 65 min. The nano ESI MS/MS was performed using a HCT Ultra ion trap mass spectrometer (Bruker). ESI was delivered using distal-coating spray Silica tip (id 20 µm, tip inner id 10 µm, New Objective, Ringoes, NJ). Mass spectra were acquired in positive ion mode, capillary voltage at −1200 V and active ion charge control trap scanning from 300 to 1500 m/z; Using an automatic switching between MS and MS/MS modes, MS/MS fragmentation was performed on the two most abundant ions on each spectrum using collision-induced dissociation with active exclusion (excluded after two spectra, and released after 2 min). The complete system was fully controlled by HyStar 3.2 software.

Mass spectra data processing was performed using Mascot Distiller (Version 2.4.3.3) with search and quantitation toolbox options. The generated de-isotoped peak list was submitted to an in-house Mascot server 2.4.0 for searching against the SwissProt database version 2013_01 (538849 sequences; 191337357 residues). Mascot search parameters were set as follows: species, *Homo sapiens* (20,233 sequences); enzyme, trypsin with maximal 2 missed cleavage; fixed modification: cysteine carbamidomethylation; variable modifications: methionine oxidation, Gln->pyro-Glu (N-term Q), Glu->pyro-Glu (N-term E), Label:13C(6)15N(2) (K), Label:13C(6) (R); 0.90 Da mass tolerance for precursor peptide ions; and 0.6 Da for MS/MS fragment ions. SILAC quantitation was performed in Mascot Distiller using SILAC K+8 R+6 quantitation method, SILAC ratios for heavy and light peptide pairs were calculated by Simpsons integration method, minimum 1 peptide with unique sequence and 0.05 of significant threshold. The following criteria were used to evaluate protein identification: one or more unique peptides with ion score ≥45 and two or more unique peptides with ion score ≥30 (*p*≤0.05 threshold); proteins identified were extracted using MS Data Miner (MDM) [26]. Quantified proteins with ≥1.5 and ≤0.7 fold change were selected and clustered by biological functions, pathway and network analysis using Ingenuity computational pathway analysis (IPA) software (www.ingenuity.com) for bioinformatics analysis.

### Western Blot Analysis

Protein samples (40 µg) were separated by 10% gradient SDS-PAGE and then transferred to a nitrocellulose membrane in a blotting chamber (BioRad) at 100 V for 30 min. The membrane was blocked with 5% powdered milk in Tris-buffer saline solution (pH 7.6) containing 0.05% Tween-20 (TBS/T) then probed with antibodies against (PKM2, HK-1, HK-2, G6PD, HIF-1α and Vpr) diluted 1∶500. Membranes were incubated with primary antibodies overnight at 4°C, washed, and then incubated with appropriate HRP-conjugated secondary antibodies at room temperature for 1 h. ECL Plus kit (GE healthcare) for HRP was used according to the manufacturer’s instructions and signals were captured onto X-ray film.

### Statistical Analysis

Statistics for IPA analysis can be found at http://www.ingenuity.com/index/html. For western blot analysis Student’s t-test was used for statistical analysis and *p* ≤ 0.05 was considered statistically significant.

## Results

### Proteome of Vpr Transduced Macrophages

**Figure 1:**
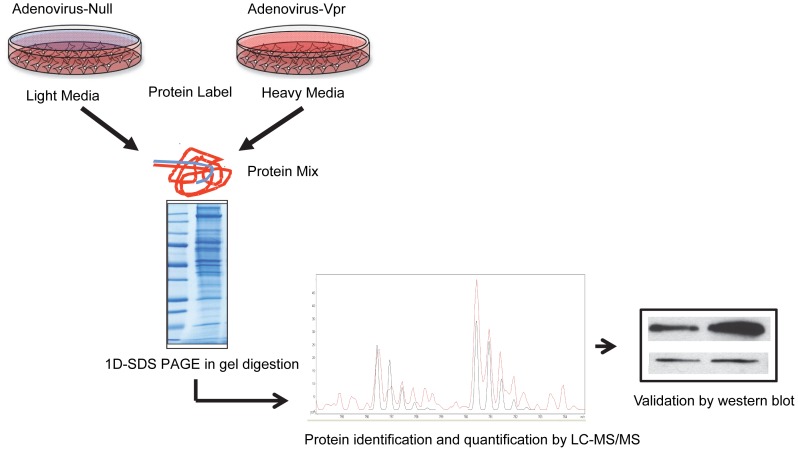
Experimental strategy for SILAC based proteomics. PMA differentiated U937 cells cultured in light or heavy media and then transduced with Adeno-Null or Adeno-Vpr virus, respectively. Protein lysates were prepared and mixed in 1∶1 ratio. Sample complexity was reduced prior to LC-MS/MS analysis by fractionation at the protein level by SDS-PAGE. Expression levels of selected proteins were validated by western blot analysis.

To get a global perspective of the molecular pathways perturbed by Vpr in macrophages, we employed SILAC in conjunction with LC-MS/MS to assess Vpr-induced differential expression of the whole proteome of U937 derived macrophages (Figure 1).

In this study, PMA differentiated U937 macrophages were cultured in both light and heavy media. The light labeled cells were transduced with Adeno-Null virus and the heavy labeled cells were transduced with Adeno-Vpr. Cell pellets prepared 72 h post transduction were lysed, and the lysates were combined and subsequently fractionated by SDS-PAGE. After in-gel digestion, the proteins were identified and quantified by LC-MS/MS. This analysis was performed once and a total of 614 proteins were identified and quantified. Details of all quantified proteins can be found in File S1).

For quantitative analysis of differences between paired experimental samples we chose a ratio of ≥1.5 or ≤0.7 as threshold for screening significantly changed proteins. Using this criterion we identified a total of 136 proteins that displayed significant changes in Vpr expressing macrophages, among which 67 and 69 were up- and down- regulated, respectively as shown in the differential protein expression list (Sup. info. 1).

### Functional Characterization of Identified Proteins and Bioinformatics Analysis

**Figure 2:**
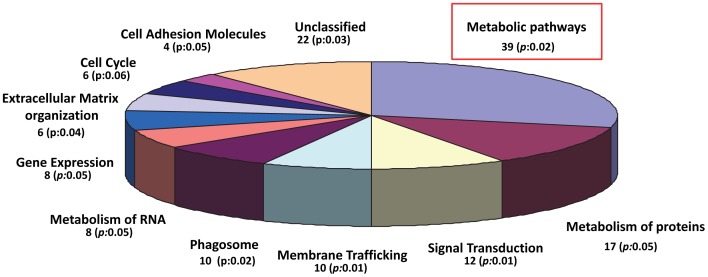
Categorization of molecular function of differentially expressed proteins in Vpr transduced macrophages. The pie graph demonstrates that among the 136 differentially expressed proteins majority of them cluster in the metabolic pathways and metabolism of proteins.

We next used the 136 differentially expressed proteins identified in response to Vpr over expression, to perform a biological function, pathway and network analysis using the Ingenuity Pathway Analysis (IPA) software. According to the molecular function analysis (Figure 2), most of the proteins were related with metabolic pathways (39%), protein metabolism (17%), cell cycle regulation (15%), signal transduction (12%), phagosomal activity (10%), membrane trafficking (10%), gene expression (8%), RNA metabolism (8%), cell cycle (6%), extracellular matrix organization (6%), signal transduction (5%), cell adhesion molecules (4%) and unclassified (22%).

**Figure 3:**
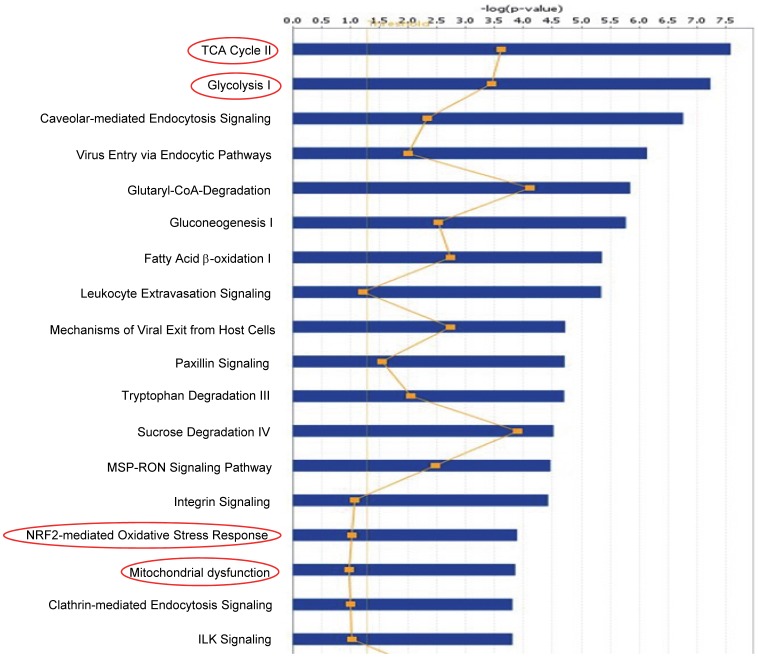
Top network functions generated using Ingenuity protein analysis for U937 cells transduced with Vpr. Graph represents host cell functions with highest score (y-axis) based on the number of differentially regulated proteins.

The list of the proteins that were altered and are related with metabolic pathway is listed in Table 1 (also listed in File S2). The canonical pathways that were identified at statistically significant levels (*p*≤0.05) are depicted in Figure 3, highlighted are the TCA cycle, glycolysis, NRF2 mediated oxidative stress response and mitochondrial dysfunction. The other pathways that were identified at statistically significant levels (p≤0.05) include virus entry via endocytic pathway, viral exit from host cells, fatty acid beta-oxidation, HIF-1 alpha signaling and others (Figure 3; also see File S2). The scores (-log [p values]) reflect the probabilities of such associations occurring by chance, with the threshold value for significance set at 1.25.

**Table 1:** Macrophage proteins within metabolic pathways altered in response to HIV-1 Vpr.

ID	Symbol	Fold change	Entrez Gene name
P51659	HSD17B4	2.69	hydroxysteroid (17-beta) dehydrogenase 4
P15586	GNS	2.50	glucosamine (N-acetyl)-6-sulfatase
P62873	GNB1	1.94	guanine nucleotide binding protein, beta polypeptide 1
Q8TC12	RDH11	1.87	retinol dehydrogenase 11 (all-trans/9-cis/11-cis)
P09211	GSTP1	1.86	glutathione S-transferase pi 1
Q9Y6N5	SQRDL	1.85	sulfide quinone reductase-like (yeast)
Q02218	OGDH	1.67	Oxoglutarate (alpha-ketoglutarate) dehydrogenase (lipoamide)
Q92945	KHSRP	1.66	KH-type splicing regulatory protein
P11413	G6PD	1.65	glucose-6-phosphate dehydrogenase
O60488	ACSL4	1.64	acyl-CoA synthetase long-chain family member 4
P07954	FH	1.59	fumarate hydratase
P36957	DLST	1.58	dihydrolipoamide S-succinyltransferase
P52789	HK2	1.52	hexokinase 2
Q13510	ASAH1	1.52	N-acylsphingosine amidohydrolase (acid ceramidase) 1
P14618	PKM	1.52	pyruvate kinase, muscle
P19367	HK1	1.51	hexokinase 1
O95831	AIFM1	−1.43	apoptosis-inducing factor, mitochondrion-associated, 1
P30101	PDIA3	−1.44	protein disulfide isomerase family A, member 3
O14880	MGST3	−1.53	microsomal glutathione S-transferase 3
O95571	ETHE1	−1.55	ethylmalonic encephalopathy 1
P09972	ALDOC	−1.55	aldolase C, fructose-bisphosphate
Q9Y2Q3	GSTK1	−1.55	glutathione S-transferase kappa 1
P05455	SSB	−1.60	Sjogren syndrome antigen B (autoantigen La)
P40939	HADHA	−1.66	hydroxyacyl-CoA dehydrogenase
P31040	SDHA	−1.66	succinate dehydrogenase complex, subunit A
P04406	GAPDH	−1.67	glyceraldehyde-3-phosphate dehydrogenase
P49448	GLUD2	−1.68	glutamate dehydrogenase 2
P60174	TPI1	−1.78	triosephosphate isomerase 1
P42126	ECI1	−1.79	enoyl-CoA delta isomerase 1
Q9H3P7	ACBD3	−1.82	acyl-CoA binding domain containing 3
P04075	ALDOA	−1.82	aldolase A, fructose-bisphosphate
P40926	MDH2	−1.93	malate dehydrogenase 2, NAD (mitochondrial)
P48735	IDH2	−2.20	isocitrate dehydrogenase 2 (NADP+), mitochondrial
Q99714	HSD17B10	−2.27	hydroxysteroid (17-beta) dehydrogenase 10
P06744	GPI	−2.31	glucose-6-phosphate isomerase
P24752	ACAT1	−2.62	acetyl-CoA acetyltransferase 1
P29401	TKT	−2.75	transketolase
Q96AB3	ISOC2	−2.78	isochorismatase domain containing 2
P54819	AK2	−3.32	adenylate kinase 2

**Figure 4:**
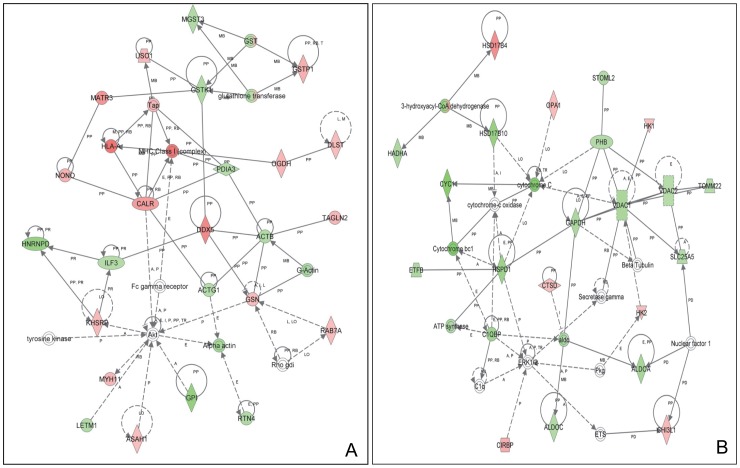
Ingenuity Pathway Analysis of proteins that were significantly altered in U937 cells transduced with Adeno-Vpr. Two relevant networks were generated from the Vpr-modulated proteins according to the Ingenuity Pathway Knowledge Criteria. **A.** Organ morphology, nucleic acid metabolism, small molecule biochemistry (score = 49). **B.** Carbohydrate metabolism, energy production, nucleic acid metabolism (score = 41). Red, up-regulated proteins; green, significantly down-regulated proteins; white, proteins known to be in the network but were not identified or identified in our study. The color depth indicates the magnitude of the change in protein expression level. The shapes are indicative of the molecular class (i.e., protein family). Lines connecting the molecules indicate molecular relationships. Dashed lines indicate indirect interactions, and solid lines indicate direct interactions. The arrow styles indicate specific molecular relationships and the directionality of the interaction.

Proteins that changed significantly in Vpr expressing macrophages were mapped to 6 specific functional networks, with each network containing 13 or more “focus” members (Table 2). The top three networks of interest correspond to (1) organ morphology, nucleic acid metabolism, small molecule biochemistry (score = 49), (2) carbohydrate metabolism, energy production, nucleic acid metabolism (score = 41), and (3) nucleic acid metabolism, small molecule biochemistry, DNA replication, recombination, and repair (score = 33). Figure 4A and B shows the “interactomes” of the top 2 (out of 9 significant scoring, Sup. Info 2) local connecting networks and functional associations within those networks.

**Table 2:** List of relevant network that were constructed by IPA analysis from the 136 Vpr-modulated proteins in macrophages.

ID	Top Functions	Score	Focus molecules	Molecules in network
1	Organ Morphology, Nucleic Acid Metabolism, Small Molecule Biochemistry	49	25	ACTB, ACTG1, Akt, Alpha actin, ASAH1,CALR, DDX5, DLST, Fc gamma receptor, G-Actin, glutathione transferase, GPI, GSN, GST, GSTK1, GSTP1,HLA-A, HNRNPD, ILF3, KHSRP, LETM1, MATR3, MGST3, MHC Class I (complex), MYH11, NONO, OGDH, PDIA3, RAB7A, Rho gdi, RTN4, TAGLN2,Tap, tyrosine kinase, USO1
2	Carbohydrate Metabolism, Energy Production, Nucleic Acid Metabolism	41	22	3-hydroxyacyl-CoA dehydrogenase, ALDOA, ALDOC, ATP synthase, Beta Tubulin, C1q, C1QBP, CHI3L1, CIRBP, CTSD, CYC1,Cytochrome bc1, cytochrome C, cytochrome-c oxidase, ERK1/2, ETFB, ETS, GAPDH, HADHA, HK1, HK2, HSD17B4, HSD17B10, HSPD1, Nuclear factor 1, OPA1, PHB, Pkg, Secretase gamma, SLC25A5, STOML2, TOMM22, VDAC1, VDAC2
3	Nucleic Acid Metabolism, Small Molecule Biochemistry, DNA Replication, Recombination, and Repair	33	20	ACTA2, Actin, adenosine-tetraphosphatase, Alpha Actinin, Alpha catenin, ATP5A1, ATP5B, ATP5D, ATP5F1, ATP6V1B2, ATPase, CaMKII, CAPG, CAPZA1, caspase, CD3, CLIC1, CLTC,F Actin, FH, G6PD, Hsp27, Lamin, Lamin b, LMNA, LMNB1, LMNB2, MDH2,Mlc, MYH9, PFN1, PI3K (complex), PLEC, Rock, VIM
4	Cancer, Gastrointestinal Disease, Hepatic System Disease	28	17	26s Proteasome, 60S ribosomal subunit, AIFM1, AK2, CD44, Ck2, EEA1, estrogen receptor, HDGF, Histone h3, Histone h4, Hsp70, Hsp90, HSP, HSP90AB1, HSPA2, HSPA5, Jnk, MTDH, NPM1, P38 MAPK, PDGFBB, PKM, PP2A, Rb, RNA polymerase II, Rnr, RPL11, RPL13, RPL29, RPLP0, Sos, SSB, trypsin, UBA52
5	Developmental Disorder, Hereditary Disorder, Metabolic Disease	27	16	ACBD3, ACTBL2, ATP6V1H, C20orf24, CTSA, ECI1, ETHE1, GLUD2, GNS, GOLGB1, ISOC2, MAPKAP1, MYO1G, NAA38, NEU1, PGRMC2, PXK, SCAMP3, SHCBP1, SLC9A3R2, SNX27, SQRDL, SRGAP2, SUCLG1, TFEB, TMEM33, TOM1L1, TP53I3, UBC, UNC93B1, USP1, USP46, VAT1, WDR20,ZUFSP
6	Cellular Development, Cellular Growth and Proliferation, Tissue Morphology	18	13	ACAT1, calpain, Cathepsin, collagen, Collagen type I, Collagen type II, Collagen type III, Collagen type IV, Collagen(s), CTSB, CTSH, DYSF, Ecm, Fibrin, Fibrinogen, Filamin, FTH1, FTL, HYOU1, Integrin, Integrin alpha 2 beta 1, Integrin α, ITGAX, Laminin, LDL, Lfa-1, MMP1, NFkB (complex), P4HB, PDGF (family), PECAM1, Rab11, RRBP1, TFRC

Proteins were named using HUGO gene nomenclature.

### Validation of Protein Identification and Quantification

**Figure 5:**
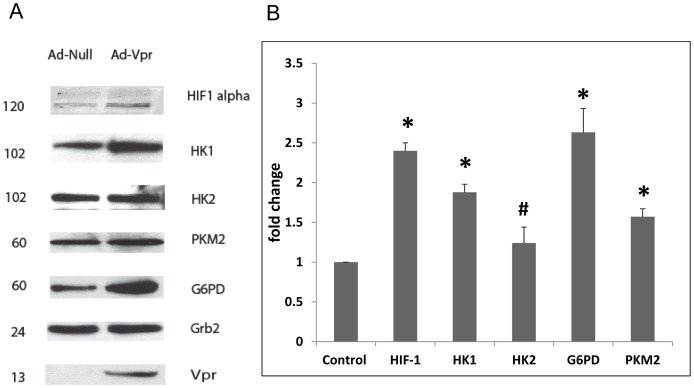
Validation of protein expression by western blot analysis. A. Western blot analysis of protein lysates prepared from macrophages derived from U973 cells transduced with Adeno-Null or Adeno-Vpr virus. Molecular weight of respective protein is shown in kDa. B. Densitometric analyses of the representative proteins were done after normalization to Grb2 levels. Values represent means ± S.E of three experiments. *indicate p value<0.05, ^#^indicate p value>0.05 or not significant.

Since the functional characterization and bioinformatics analysis revealed that the pathways that were significantly altered involved glycolysis, mitochondrial dysfunction, and HIF-1α signaling, we therefore focused on the validation of relative abundance of some of the proteins in these pathways by western blot analysis. The western blot analysis demonstrates that the changes in the ratios of representative proteins (HK-1, HK-2, PKM2, G6PD), between adeno-null transduced and adeno-Vpr transduced macrophages (Figure 5A and B) are consistent with that derived from SILAC studies. In tune with our earlier observation that Vpr induces HIF-1 alpha [27] we have observed a significant increase in HIF-1 alpha levels in Vpr transduced macrophages. In contrast, Figure 5A, shows data for Grb2 (Growth factor receptor-binding protein 2) protein remained unchanged between null and Vpr transduced cells. Taken together, these data indicate that the SILAC strategy can efficiently detect specific protein alterations in macrophages that overexpress HIV-1 Vpr.

## Discussion

Over the last few years, proteomics has contributed significantly in HIV research to investigate not only HIV pathogenesis but also for identification of potential biomarkers [28]–[35]. In this study, we utilized the high-throughput quantitative proteomic approach using SILAC to obtain information about the macrophage proteome in the context of Vpr-host interaction since Vpr from strain 89.6 demonstrates PP2A-dependent apoptosis in CD4^+^ T cells and Vpr gene polymorphism is known to influence clinical outcomes [19], [36]. A total of 136 different proteins were identified as having altered abundances in Vpr transduced macrophages, including those involved in pyruvate metabolism, pentose phosphate pathway, mitochondrial dysfunction, oxidative stress, HIF-1 alpha signaling, and cell cycle: G2/M DNA damage checkpoint regulation. Previous reports have demonstrated the effects of HIV-1 on metabolic and neurological pathways at the level of transcriptome in diverse cell types including the brain [37]–[41]. Our proteomics data are in concordance with the transcriptome analysis that also demonstrated the interference of HIV-1 in host energy metabolism pathways [38], [41].

In this study we have found increased expression of numerous enzymes involved in glycolytic and citrate pathways viz., Hexokinase (HK), Glucose-6-phosphate dehydrogenase (G6PD), pyruvate kinase M2 (PKM2), and Fumarate hydratase (Fumarase).

**Figure 6:**
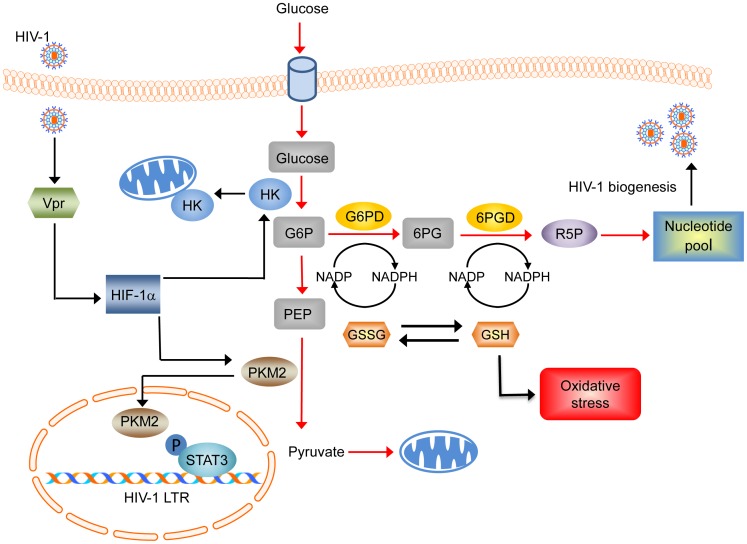
Schematic representation of the mechanism of regulation of glycolysis by HIV-1 Vpr in macrophages. HIV-1 Vpr induces HIF-1α that in turn induces HK and PKM2. Upregulation of HK expression on one hand results in conversion of glucose into glucose-6-phosphate (G6P) and forward feed into phosphoenolpyruvate (PEP) for conversion into pyruvate by PKM2 which then enters into mitochondria for conversion into Acetyl-CoA via the TCA cycle. On the other hand, HK can translocate to the outer mitochondrial membrane to confer an anti-apoptotic phenotype. Glucose, in addition can be metabolized by upregulation of Glucose-6-phosphate dehydrogenase (G6PD) via the pentose-phosphate pathway (PPP). In this pathway glucose-6-phosphate is decarboxylated to form ribose-5-phosphate (R5P) in a series of reaction involving 6-phosphogluconate dehydrogenase (6PGD) for the synthesis of nucleotides. The pentose phosphate pathway also reduces NADP+ to NADPH (H+), a cofactor necessary for antioxidant glutathione (GSH) regeneration from its disulfide form (GSSG) for attenuation of oxidative stress. Nuclear translocation of PKM2 can lead to Stat3 phosphorylation and activation of HIV-1 LTR.

HK is the rate-limiting enzyme that converts glucose in to glucose-6-phosphate (G6P), while G6PD a member of the pentose phosphate pathway (PPP) is involved in the conversion to G6P to 6PG [42]. Furthermore, upregulation of hexokinase expression and shunting of glucose through the pentose phosphate pathway (PPP) creates a restrictive environment for cytochrome *c*-mediated apoptosis as a result of increased translocation of HK to outer mitochondrial membrane (OMM), and generation of NADPH, respectively (Figure 6) [43], [44]. Cellular redox status is maintained by scavenging ROS by glutathione whose synthesis is regulated by NADPH [44]. In addition, upregulation of PPP and activation of G6PD also promotes nucleotide biosynthesis that provides the nucleotide pool [45] required for sustained HIV-1 replication (Figure 6).

PKM2 acts as a glycolytic enzyme, transferring a phosphate group from phosphoenolpyruvate (PEP) to ADP to yield one molecule of pyruvate and one molecule of ATP [42]. Earlier studies from our laboratory have demonstrated the role of oxidative stress in Vpr mediated regulation of HIF-1 alpha [27]. Interestingly, HIF-1 alpha not only induces PKM2 expression but also other enzymes involved in glucose metabolism [46]. Earlier studies have demonstrated upregulation of PKM2 in HIV-1 infected macrophages and in HIV-1 infected human astrocytes treated with cocaine [47], [48] however, its role in HIV-1 LTR activation remains unknown. Recent studies demonstrate that PKM2 has non metabolic functions and plays significant role in regulating gene transcription [49], [50].

PKM2 is apparently a dual-specificity protein kinase since it phosphorylates Stat3 at Y705 and histone H3 at T11 [49], [50]. Furthermore, phosphorylation of H3-T11 by PKM2 leads to the induction of Myc gene transcription following dissociation of HDAC3 [50]. Interestingly, HDAC3 inhibition leads activation of latent HIV-1 [51]. HIV-1 (BaL) and HIV-1 (LAI) are known to selectively induce phosphorylation of Stat3, and repression of Stat3 expression inhibits HIV-1 replication [52]. Based on these observations, it can be proposed that Vpr mediated PKM2 up regulation might play a role in Stat3 mediated HIV-1 LTR activation or HDAC3 dissociation from HIV-1 LTR in HIV-1 latency. Future studies are to be undertaken to elucidate the proposed mechanism.

Fumarate hydratase (Fumarase) is member of the tricarboxylic acid (TCA) cycle in mitochondria and converts fumarate to malate [53]. The cytosolic isoform of fumarase plays a role in DNA repair by translocating from the cytoplasm to nucleus [54].

The upregulation of PKM2, G6PD and fumarate hydratase in our study are in tune with an earlier study that demonstrated increased levels of pyruvate, NADPH and malate in the metabolite pool of activated U1 cells (HIV-1 infected U937 cells) by LC-MS/MS analysis [55]. Interestingly, some of the other proteins that are upregulated are transferrin receptor-1 (TfR1), hypoxia up-regulated protein 1 (HYOU1) also known as oxygen-regulated protein 150 (ORP-150), USO1 vesicle docking protein homolog (USO1), matrin 3, and glutathione S-transferase Pi 1. Among these proteins upregulation of HYOU1 and USO1 was demonstrated in HIV-1 infected CD4^+^T cells [33].

TfR1 is a cell membrane-associated glycoprotein responsible for incorporation of the iron bound to transferrin through an endocytotic process from the circulating blood [56]. Interestingly, TfR1 is upregulated by HIF-1 activation [57]. HYOU1/ORP-150 belongs to heat shock protein 70 family and plays an important role in protein folding and secretion in the ER [58], [59]. Suppression of HYOU1/ORP150 expression leads to accelerated apoptosis [60]. Physical interaction between HIV-1 gp120 and HYOU1 was observed in human HEK293 and/or Jurkat cell lines by using affinity tagging and purification mass spectrometry analyses [61]. USO1 also known as Golgi complex-associated protein p115 is known to play a critical role in the regulated secretion of macrophage migration inhibitory factor (MIF) from monocytes/macrophages [62]. Matrin 3 has been shown to bind Rev/RRE-containing viral RNA and stabilize unspliced and partially spliced HIV-1 transcripts that subsequently results in increased cytoplasmic expression of these viral RNAs [63]. Glutathione S-transferases (GST) are involved in cellular protection against oxidative stress. Transcriptional induction of GSTP1 has been shown to be a part of an adaptive response to oxidative stress [64], and also to protect 3T3 mouse fibroblasts against H_2_O_2_ mediated oxidative stress [65] and is postulated to be involved in signaling during oxidative stress.

Among the proteins whose expression is down regulated in Vpr transduced macrophages are Glutamate dehydrogenase 2 (GLUD2), Adenylate kinase 2 (AK2) and Transketolase (TKT). GLUD2 is a mitochondrial enzyme involved in glutamate metabolism it catalyzes the reversible oxidative deamination of glutamate to alpha-ketoglutarate [66]. Dysregulation of glutamate metabolism in macrophages therefore can contribute to neurodegeneration via neuroexcitotoxic mechanisms in the context of NeuroAIDS. AK2 is a mitochondrial enzyme that regulates adenine nucleotide interconversion [67]. AK2 is known to mediate mitochondrial apoptosis through the formation of an AK2-FADD-caspase-10 (AFAC10) complex [68]. Thus, down regulation of AK2 by Vpr might play an anti-apoptotic role in macrophages. TKT is a thiamine-dependent enzyme that plays a role in the channeling of excess sugar phosphates to glycolysis in the pentose phosphate pathway [69].

### Conclusions

We have used a SILAC-based proteomic approach to investigate alteration in the macrophage proteome by HIV-1 protein Vpr. Our studies demonstrate how a single viral protein as opposed to the whole virus has comparable impact and underscores the role of Vpr in modulating changes at the transcriptome and proteome level in HIV-1 infected host. It would be interesting to further investigate the role of the identified proteins, specifically PKM2 in HIV-1 replication, and HK and G6PD in anti-apoptotic pathways in macrophages. We postulate that HIV-1 hijacks the macrophage glucose metabolism pathway via the Vpr-HIF-1 alpha axis to create an environment that is not only advantageous for viral replication and biogenesis, but also for long-term survival of infected macrophages (Figure 6). We anticipate that the information obtained from this and future studies would enhance our understanding of the role of Vpr in HIV-1 replication and may help formulate novel therapeutic approaches that targets glucose metabolism to mitigate HIV replication and survival in macrophages.

## Supporting Information

File S1 (XLS)

File S2 (XLS)
